# TRP Channels: The Neglected Culprits in Breast Cancer Chemotherapy Resistance?

**DOI:** 10.3390/membranes13090788

**Published:** 2023-09-12

**Authors:** Mayar Soussi, Alice Hasselsweiller, Dimitra Gkika

**Affiliations:** Univ. Lille, CNRS, Inserm, CHU Lille, UMR9020-U1277—CANTHER—Cancer Heterogeneity Plasticity and Resistance to Therapies, F-59000 Lille, France; mayar.soussi.etu@univ-lille.fr (M.S.); alice.hasselsweiller.etu@univ-lille.fr (A.H.)

**Keywords:** breast cancer, TRP channels, chemotherapy, chemoresistance taxanes, anthracyclines, tamoxifen

## Abstract

Breast cancer is a major health concern worldwide, and resistance to therapies remains a significant challenge in treating this disease. In breast cancer, Transient Receptor Potential (TRP) channels are well studied and constitute key players in nearly all carcinogenesis hallmarks. Recently, they have also emerged as important actors in resistance to therapy by modulating the response to various pharmaceutical agents. Targeting TRP channels may represent a promising approach to overcome resistance to therapies in breast cancer patients.

## 1. Introduction

Cancer is a complex and heterogeneous disease that affects millions of individuals worldwide. In the face of notable progress in unraveling their molecular basis and the development of innovative treatments, an intriguing fact is emerging in the clinic: a subset of patients faces recurrence of the disease, leading to treatment failure [1]. At the core of this challenge lies an obstacle that compromises the efficacy of chemotherapeutic treatment [2]. Chemoresistance manifests itself via two distinct mechanisms. Firstly, tumors may possess intrinsic resistance mechanisms even before chemotherapy initiation, making treatment less effective from the beginning. Secondly, tumors that have proven sensitive to chemotherapy may potentially develop resistance as treatment progresses, further complicating the therapeutic trajectory [3]. Hence, understanding the mechanisms underlying chemoresistance is of great importance in the pursuit of effective approaches for cancer therapy [4].

**Figure 1:**
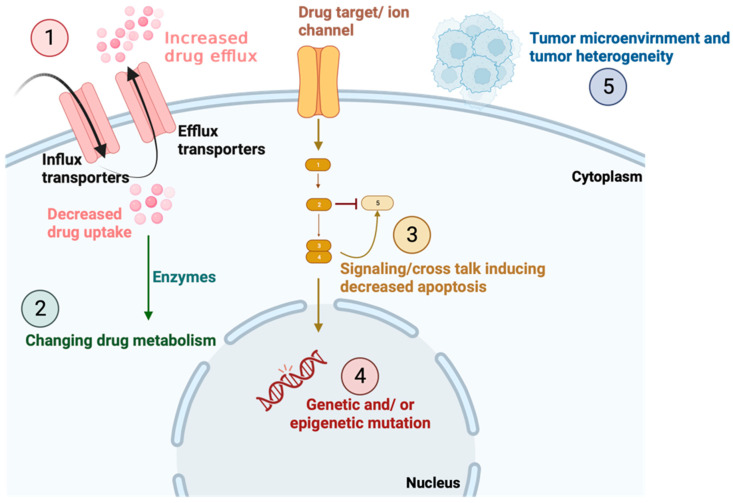
Drug resistance mechanisms in cancer cell. Drug resistance can arise via diverse mechanisms. These include (1) the enhancement of drug efflux via membrane transporters and the reduction in drug uptake via influx transporters, (2) the increase and changes in drug metabolism, (3) the mutations in drug targets or the activation of other targets and signaling pathways leading to the inhibition of apoptosis, (4) the increase in cell adaptability via epigenetic and miRNA regulation, and (5) the changes in the microenvironment, such as hypoxia response.

Numerous mechanisms can lead to drug resistance in cancer (Figure 1). For instance, cancer cells can develop chemoresistance by elevating the efflux of drugs from within the cells via ATP-binding cassette (ABC) transporters and/or reducing the absorption of the drugs via influx transporters, such as solute carriers [5,6]. Significant evidence strongly suggest that cancer cell resistance to a wide range of anticancer drugs, including anthracyclines, is mediated by the overexpression of ABCC1, which is the first ABC transporter identified in humans [7]. Additionally, the acquisition of chemoresistance could be achieved via changes in drug metabolism, such as reducing the activation of drugs and increasing drug inactivation with the help of enzymes such as glutathione S-transferase and cytochrome P450 [8,9]. Indeed, these enzymes regulate the levels of the agents in both the intracellular and extracellular compartments [10]. In addition, the effectiveness of chemotherapeutic agents can be affected by mutations and alterations in the expression levels of the molecules they target [10]. For example, cancer cells with mutant topoisomerase II may alter the cell’s response to doxorubicin [11]. Additionally, epigenetic changes and the regulation of miRNA are fundamental mechanisms that contribute significantly to the development of drug resistance in cancer treatment. It has been shown that the demethylation of the multi-drug resistance (MDR1) gene in cancer cells leads to chemoresistance caused by the reduced accumulation of anti-tumor agents within the cancer cells [10]. Likewise, the demethylation of the hMLH1 promoter gene, a key gene involved in DNA mismatch repair, via decitabine treatment coupled with the restoration of the mismatch repair system, enhances the sensitivity of colorectal cancer cells to fluorouracil-5 [12]. Recent studies focusing on miRNA profiling have also provided evidence supporting the significant role of these small molecules in the development of chemosensitivity or chemoresistance [10]. Moreover, alterations in the apoptotic signaling pathways can lead to drug resistance by interfering with apoptosis induction in response to cellular stress and DNA damage. For example, mutations in the p53 gene have the potential to disrupt the link between DNA damage caused by chemotherapeutic agents and apoptosis initiation, thereby affecting the effectiveness of cancer treatment [13]. Finally, alterations in the microenvironment, such as hypoxia (low oxygen levels), can trigger the onset of chemoresistance response and tumor heterogeneity. Hypoxia contributes to chemoresistance in several crucial ways. Insufficient vasculature limits drug penetration into the affected areas [14]. Additionally, hypoxia creates an acidic tumor microenvironment, hindering the uptake of certain drugs [15]. Some drugs’ cytotoxic effects depend on oxygen availability, reducing their efficacy under hypoxic conditions [16]. Hypoxia can also act as a barrier to immune cells, influencing tumor immunity [17].

In the battle against chemoresistance, understanding these mechanisms is vital for developing effective cancer therapies. By unraveling the complexities of chemoresistance, researchers and clinicians can pave the way towards overcoming this challenge and improving outcomes for cancer patients worldwide.

Breast cancer is the most prevalent form of cancer diagnosed among women worldwide. It is a significant health concern and the primary cause of cancer-related deaths [18]. Currently, chemotherapy is the standard and systemic treatment option for it. Conversely, breast cancer cells frequently exhibit elevated levels of the ion channel expression. Certain studies have noted a link between the expression levels of these channels, notably for five TRP channels, and resistance to the cytotoxic drugs employed in breast cancer chemotherapy regimens.

Within this review, we will assess the existing understanding of how five specific TRP channels (TRPA1, TRPC5, TRPV1, TRPV2, and TRPM2) contribute to the resistance of breast cancer to therapeutic interventions. Additionally, we will explore the potential utility of targeting TRP channels as an innovative therapeutic approach aimed at surmounting this chemotherapy resistance.

## 2. Breast Cancer Chemotherapy: Decoding the Enigma of Resistance

Breast cancer stands as the most frequently diagnosed cancer among women and holds the position of being the primary cause of cancer-related deaths [18]. It is tackled via a comprehensive approach that embraces the power of multidisciplinary treatments. This encompassing strategy involves a diverse array of therapies, including neoadjuvant chemotherapy surgery for operable tumors, adjuvant chemotherapy, radiation, and hormonal and targeted therapies [19]. By combining these modalities, healthcare professionals are working to optimize treatment outcomes and provide patients with the best possible care. In breast cancer, chemotherapy stands as the primary systemic treatment modality [20], triggering tumor cell death and reducing tumor burden. The choice of the specific chemotherapy regimen relies on many factors such as the stage of the disease and the patient’s individual characteristics. Some of the most studied chemotherapeutic agents include anthracyclines, taxanes, and platinum-based drugs [19].

### 2.1. Anthracyclines

Anthracyclines, such as doxorubicin and epirubicin, are known for their effectiveness in the treatment of early-stage breast cancer due to their antineoplastic effects. Their molecular mechanism of action involves DNA base pairs’ intercalation, leading to the stabilization of the topoisomerase IIα complex and resulting in increased DNA breaks [21], which, in turn, prevent DNA and RNA synthesis [22]. The emergence of resistance in tumor cells towards anthracyclines poses a significant challenge in cancer treatment. In general, the mechanisms of anthracycline resistance are multifactorial and involve changes in membrane transport and an altered drug efflux mediated by ABC superfamily proteins, such as p-glycoprotein (P-gp), Multidrug Resistance Protein 1 (MRP1), Lipoprotein Receptor-related Protein 1 (LRP), and Breast Cancer Resistance Protein (BCRP). For instance, an inverse correlation was shown between the MDR1 expression in breast tumors and the effectiveness of first-line chemotherapy containing anthracyclines [23]. Additionally, alterations in p53, which is a tumor suppressor, can affect the cytotoxicity of anthracyclines. For example, in fibroblast models, the absence of p53 results in doxorubicin resistance and diminishes the extent of apoptotic cell death induced by doxorubicin [24]. Another potential mechanism implicated in the development of resistance to anthracyclines includes changes in cellular glutathione levels, decreased activation of doxorubicin by cytochrome-P450-reductase, and NADPH resulting from alterations in NADPH metabolism [25]. Additionally, the sensitivity of cells to anthracyclines was proven to be dependent on topoisomerase II’s gene expression and activity [26].

### 2.2. Taxanes

Another drug family pivotal in metastatic breast cancer chemotherapy is the taxanes, such as paclitaxel and docetaxel [27]. Taxanes act by stabilizing microtubules, leading to cell cycle arrest and abnormal mitosis. They disrupt centrosomal function, leading to the impaired formation of normal spindles, and they suppress the dynamics of spindle microtubules. These actions result in the blockage of cell cycle progression. The induction of apoptosis can occur via two mechanisms: aberrant mitosis or the development of a multinucleated G1-like state due to mitotic slippage. However, the occurrence of these events and their reliance on cell type and the drug administration schedule can vary [27].

To acquire resistance to taxanes, treated cancer cells change the composition of microtubules, favoring isoforms that are less susceptible to the effects of taxanes. Moreover, modifications in the signaling mechanisms of the mitotic checkpoint can reduce the effectiveness of taxanes, thereby hindering aberrant mitotic advancement and the occurrence of apoptosis [28].

### 2.3. Platinum-Based Drugs

In the same regard, we can also mention Platinum-Based Drugs, which are cisplatin, carboplatin, oxaliplatin, nedaplatin, and lobaplatin [28,29], and their promising role in treating breast cancer, especially those with genetic mutations in the BRCA1 gene. Their mechanism of action involves binding to DNA and disrupting DNA replication, leading to impaired cell division and the induction of apoptosis in cancer cells [29]. Resistance to platinum agents remains a significant challenge, impeding their clinical efficacy. The key mechanisms encompass the altered cellular platinum accumulation, increased use of detoxification systems of the drugs, enhanced DNA repair capabilities, and diminished amount of apoptosis [30].

In addition to chemotherapy, existing clinical drugs focus on inhibiting estrogen synthesis since approximately 80% of breast tumors exhibit estrogen receptor expression. Indeed, tamoxifen, which belongs to the Selective Estrogen Receptor Modulators (SERMs), has a high effectiveness in the treatment of estrogen receptor-positive breast cancer. However, a significant challenge arises as more than 30% of estrogen receptor-positive tumors exhibit intrinsic resistance to tamoxifen therapy [31]. Many studies were conducted to understand the intricate mechanisms underlying tamoxifen resistance. This effort has led to the discovery of the complex pathways involved, counting the modulation of estrogen receptor signaling, the upregulation of growth factor receptors such as Human Epidermal Growth Factor Receptor-2 (HER2), Epidermal Growth Factor Receptor (EGFR), Fibroblast Growth Factor Receptor (FGFR), and Insulin-like Growth Factor 1 Receptor (IGF1R), as well as alterations in the PI3K-PTEN/AKT/mTOR pathway and Nuclear Factor-kappa B (NFκB) signaling. Notably, several key molecular pathways, including Mitogen-Activated Protein Kinases (MAPK) and protein kinase A, have been implicated in tamoxifen resistance [32]. These pathways induce the phosphorylation of the estrogen receptors or its co-regulatory molecules, contributing to the development of resistance [33].

## 3. TRP Channels

Transient Receptor Potential (TRP) channels encompass a set of cationic channels capable of reacting to a diverse array of stimuli. These stimuli include both the internal and external chemical mediators, as well as physical factors like mechanical force (mechanosensitive) and temperature (thermosensitive) [34]. They are also involved in a wide range of physiological and pathological functions, including the perception of pain, vision, taste, and inflammation. Most TRP channels are identified as nonselective cation channels, which are able to conduct both single-charged (monovalent) and double-charged (divalent) cations [35]. In mammals, TRP channels are classified into six subfamilies based on their primary sequences, including TRPA (ankyrin), TRPC (canonical), TRPM (melastatin), TRPV (vanilloid), TRPML (mucolipin), and TRPP (polycystin). The architecture of TRP channels shares similarities with other ion channels, consisting of a six-transmembrane helix topology (S1 through S6) with a reentrant loop between S5 and S6 that forms the channel pore [36].

New data suggest that TRP channels may play a crucial role in mediating chemotherapy resistance in breast cancer. They have become key players in cancer therapy over the past decade. Alterations in the expression and function of TRP channels are closely related to the resistance or susceptibility of cancer cells to apoptosis-induced cell death, resulting in cancer-promoting effects or the resistance to chemotherapeutic treatments. Moreover, numerous publications have highlighted the direct or functional interactions between TRP channels, particularly the canonical (TRPC) subfamily, and ORAI channels and their activator STIM. The tight interaction between ORAI and TRPC channels may serve for the activation of TRPC channels in response to elevated intracellular Ca^2+^ ([Ca^2+^]_i_) [37]. For instance, it has been shown that cisplatin resistance in the ovarian cancer cell line IGROV1 was correlated with an overexpression of TRPC1, Orai1, and Orai2. Pharmacological inhibition with SKF-96365, YM-58483, or 2-APB sensitized chemoresistant IGROV1 cells to chemotherapeutic agents [38].

Comprehending the function of TRP channels in cancer could unveil new therapeutic opportunities to overcome chemotherapy resistance and enhance the effectiveness of cancer treatments.

## 4. How Does TRP Channels Modulate Chemotherapy Resistance in Breast Cancer?

The modulation of chemotherapy resistance in breast cancer via TRP channels is a captivating phenomenon that unveils a remarkable interplay between cellular pathways. Let us embark on a journey through the intricate mechanisms by which TRP channels influence the resistance to chemotherapy in breast cancer.

### 4.1. TRPA1

The TRPA1 channel is expressed in specific primary nociceptive neurons and it serves as a sensory receptor for certain pungent compounds derived from plants such as allyl isothiocyanate (AITC), cinnamaldehyde, and allicin [39]. Of the TRP channels that detect oxidative signals, TRPA1 exhibits the most pronounced sensitivity to oxidation [40] and also knows to amplify the oxidative stress [41]. Indeed, one of the major groups of factors activating the channel in the tumor context is a variety of oxidative stress metabolites, such as reactive oxygen species (ROS) [39]. Over recent years, there have been reports indicating that cancer cells develop their defense system against oxidative stress in response to stressful environments during tumor development [42]. Moreover, this defense system is tightly associated with drug resistance during cancer treatment. Indeed, Takahashi et al. have demonstrated that TRPA1 increases intracellular calcium ([Ca^2+^]_i_) levels and cell survival in triple-negative breast cancer cells (HCC1569) upon treatment with hydrogen peroxide (H_2_O_2_), which is an activator of the TRPA1 channel [43,44].

Regarding the action of chemotherapeutic drugs on TRPA1, it has been shown that channel inhibition decreases tumor growth and increases sensitivity to carboplatin in vivo [43]. In terms of the involved signaling pathways, the presence of high levels of [Ca^2+^]_i_ activated anti-apoptotic pathways and the increased resistance to ROS was shown [43]. Thus, inhibiting the excessive opening of TRPA1 disrupts the oxidative stress defense system acquired by breast cancer cells. In fact, TRPA1 inhibition in HCC1569 cells resulted in the decreased phosphorylation of RAS-ERK/AKT/mTOR signaling proteins [43], which are the chief mechanisms for controlling cell survival and drug resistance [45].

Interestingly, a dual receptor-targeting and size-switchable “cluster bomb” was designed and used in triple-negative breast cancer cells in vitro and in vivo [46]. This cluster bomb co-loaded doxorubicin and TRPA1 inhibitor AP-18 (DA-tMN), targeting the deepest tumor sites where ROS are supposed to be elevated. By inhibiting TRPA1, the efficacy of doxorubicin chemotherapy was improved by blocking the Ca^2+^ influx induced by the drug. Additionally, TRPA1 inhibition was found to regulate EMT-related proteins, inhibiting the EMT process, which may be a possible antimetastatic mechanism [46]. These findings highlight the potential of targeting TRPA1 in combination with chemotherapy as a therapeutic strategy for breast cancer treatment, particularly in triple-negative breast cancer cases where effective treatment options are limited.

### 4.2. TRPC5

It was suggested that the TRPC5 channel may be involved in breast cancer migration and metastasis. It was shown that Rac1, which is an actin cytoskeleton regulator [47] and which is found to be overexpressed in breast tumor cells [48], is activated by a TRPC5-mediated Ca^2+^ influx [48], which promotes breast cancer cells’ migration.

**Figure 2:**
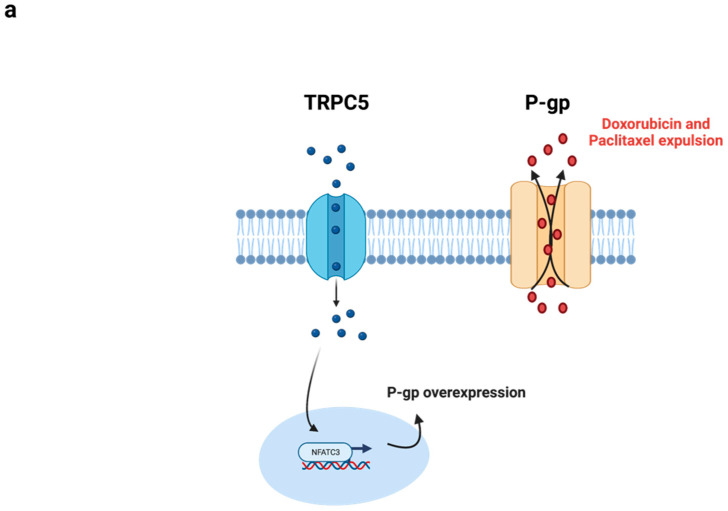

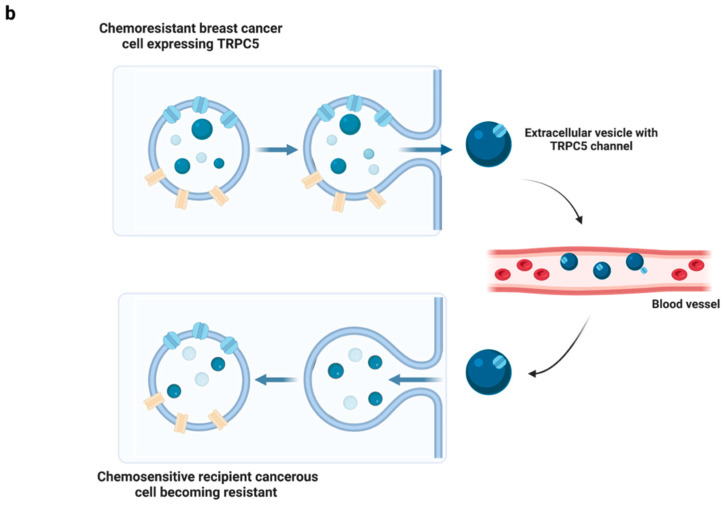
Two distinct TRPC5 mediated the cancer drug resistance. (**a**) TRPC5 overexpression activates the transcription factor NFATC3 Ca^2+^ signaling pathway, leading to p-gp overexpression. Moreover, the overexpressed p-gp expels chemotherapeutic drugs triggering chemoresistance. (**b**) Chemoresistant breast cancer cells overexpressing TRPC5 transfer channel units to chemo sensitive recipient cells via extracellular vesicles (EV), leading to the development of TRPC5-mediated chemoresistance in these cells.

In addition to its involvement in breast cancer metastasis, a positive correlation was shown for this channel with drug resistance [48]. In human breast cancer cells, the high expression of TRPC5, and consequently, the high Ca^2+^ influx via the channel activated the transcription factor NFATC3 (Nuclear Factor of Activated T Cells, Cytoplasmic 3), which triggers p-gp transcription. The overexpression of p-gp is widely recognized as a major factor in chemoresistance in cancer cells, as it functions as an active efflux pump that can remove various foreign substances, including chemotherapeutic agents, from within the cell [49] (Figure 2a). Xin Maa et al. have demonstrated that the increase in TRPC5, associated with p-gp in MCF-7 doxorubicin-resistant breast cancer cells, promotes chemoresistance. Moreover, the inhibition of the channel by several means, such as the administration of a TRPC5-specific blocking antibody (T5E3), and the overexpression of a dominant negative TRPC5 (TRPC5-DN), TRPC5 siRNA, or application of a TRP channel inhibitor (2-APB) resulted in a significant decrease in doxorubicin IC50 values. This study demonstrated not only a remarkable reversal of doxorubicin resistance in vitro and in vivo, but also changes in drug distribution leading to an increase in the doxorubicin concentration in the nucleus [50]. Interestingly, treating MCF-7 resistant cells with T5E3 and TRPC5-DN reversed the resistance to paclitaxel, as demonstrated by the MTT assay [50].

Additionally, it has been suggested that microRNA-320a (miR-320a), which acts as a tumor suppressor, could target and degrade TRPC5 and NFATC3 mRNAs, which are involved in breast cancer chemoresistance [51]. Furthermore, autophagy is frequently elevated in cancer cells and has been linked to the emergence of drug resistance [52]. In this regard, Zhang et al. have shown that chemotherapy-induced autophagy is regulated via TRPC5 in MCF-7 cells, and thus, the suppression of TRPC5 or the suppressing autophagy enhances the responses of MCF-7 cells to doxorubicin via the CaMKKβ/AMPKα/mTOR pathway [53].

Moreover, TRPC5 has been identified as a potential exosome biomarker in breast cancer chemoresistance, affecting the neighboring cells. It has been shown that chemosensitive breast cancer cells can acquire a chemoresistant phenotype when exposed to extracellular vesicles (eVs) expressing TRPC5 [54] (Figure 2b). These eVs are constantly released by certain chemoresistant breast cancer cells and have the ability to convey their drug-resistant characteristics to adjacent cells. By transferring TRPC5, recipient cells were able to produce this Ca^2+^-permeable channel, which in turn stimulated the production of p-gp in recipient cells [54]. As a result, non-resistant cells were able to acquire chemoresistance.

Overall, TRPC5 plays a significant role in breast cancer drug resistance by regulating p-gp expression, promoting autophagy, and facilitating the transfer of chemoresistance to neighboring cells via eVs. Targeting TRPC5 and its associated pathways could be a potential therapeutic strategy to overcome chemoresistance in breast cancer.

### 4.3. TRPV1

TRPV1 is a highly selective cation channel that is frequently detected in a variety of tissues [55]. It is known for its role in sensing and responding to heat, and it responds to various stimuli such as oxidative stress or capsaicin and is activated by melatonin, a hormone produced by the pineal gland in the brain. Its activity can also be modulated via capsazepine, which is a competitive antagonist of capsaicin [56,57]. The evidence for a differential TRPV1 expression has been demonstrated in a variety of tumor types, including human breast cancer cell lines. This was further elucidated by Weber et al., who demonstrated an increased TRPV1 expression in malignant breast cancer, compared with adjacent healthy tissue. It has also been shown that the combination of melatonin and doxorubicin leads to anti-tumoral effects by modulating TRPV1 channel activity [56]. In MCF-7 cells treated with doxorubicin, the [Ca^2+^]_i_ levels were elevated, but when melatonin was administered, the [Ca^2+^]_i_ levels were reduced. Additionally, various factors associated with cellular damage such as ROS and mitochondrial membrane depolarization were observed to be higher in both the doxorubicin and melatonin groups, as opposed to the control group [56]. Furthermore, the combination of doxorubicin and melatonin significantly increased apoptosis via caspase 9 [56]. TRPV1 activation was also shown to enhance both apoptosis and cisplatin oxidant effects. Gokhan Nur et al. demonstrated that cisplatin induces mitochondrial ROS production and initiates cell death in MCF-7 cells primarily via the activation of the TRPV1 channel [58]. Taken together, the anti-tumoral properties of TRPV1 can be further enhanced via chemotherapeutic drugs in breast cancer, such as doxorubicin and cisplatin. These agents modulate the calcium flux via TRPV1 and induce cell death via the intrinsic apoptotic signaling pathway. They can be taken in combination with another TRPV1 activator (such as melatonin) to further enhance the anti-tumoral effect mediated by TRPV1. Further, the pharmacology of the TRPV1 channel is well established, with a deep understanding of the channel structure, activation mechanisms, and interactions with various ligands. This knowledge is encouraging to design and development of selective modulators that can promote TRPV1 activity for therapeutic purposes.

### 4.4. TRPV2

TRPV2 can be activated at high temperatures [59]. In certain cell types, it can potentially be expressed in the endoplasmic reticulum, and its functions could extend to mechanosensing activities [60]. TRPV2 has the potential to serve as a novel biomarker for patients with triple-negative breast cancer and basal-type breast cancer. Moreover, the expression of TRPV2 improves recurrence-free survival in breast cancer patients who have undergone chemotherapy treatment [61]. For instance, it was shown that the activation of the TRPV2 channel stimulates calcium entry and the migration of MDA-MB-231 and MCF-7 cells [62]. In addition, patients with a higher TRPV2 expression demonstrated a notably improved recurrence-free survival compared to those with a lower TRPV2 expression, particularly among individuals who underwent chemotherapy as part of their treatment [63]. Elbaz et al. demonstrated how the activation of the TRPV2 channel via its agonist cannabidiol significantly increases the cellular uptake of doxorubicin, thus enhancing the sensitivity of triple-negative breast cancer cells to chemotherapy in vitro and in vivo [63]. In addition, cells treated with both doxorubicin and cannabidiol show significantly higher apoptotic signals. Indeed, cannabidiol upregulates the levels of cleaved caspase 3 and Poly ADP Ribose Polymerase (PARP) when co-administered with chemotherapy [63]. These findings highlight the potential of targeting TRPV2 as a strategy to improve outcomes for triple-negative breast cancer patients.

### 4.5. TRPM2

In noncancerous cells, the activation of the TRPM2 channel results in an influx of calcium, promoting apoptosis [64]. However, TRPM2 has a distinct role in cancer cells. Inhibition of TRPM2 or RNA interference (RNAi) targeting TRPM2 reduced proliferation and increased DNA damage in breast adenocarcinoma cells, while having no significant effect on non-cancerous breast epithelial cells [65]. There was little research in the literature that has explored the chemotherapeutic effects resulting from TRPM2 inhibition or RNAi silencing in breast cancer cells. For instance, Koh et al. demonstrated that TRPM2 inhibition by 2-APB results in increased cell death after treatment of MDA-MB-231 and MCF-7 cells with doxorubicin and tamoxifen [66]. Similarly, in MCF-7 cells, the inhibition of TRPM2 has been found to enhance the levels of DNA damage when combined with doxorubicin treatment. Interestingly, the rise in cell death caused by both TRPM2 inhibition and chemotherapy was observed to be unrelated to autophagy or caspase-independent cell death mechanisms [66]. Indeed, in this study, when the MDA-MB-231 cells were treated with doxorubicin and after TRPM2 inhibition with ACA (TRP channel blocker) the cell death level was 59% in the presence of Q-VD-Oph, which is a pan-caspase inhibitor, and this level did not decrease without Q-VD-Oph. The same results were obtained when the cells were pretreated with 3-methyladenine, an autophagy inhibitor. Although these results are not entirely clear on the signaling pathways initiating cell death flowing chemotherapy and TRPM2 inhibition, they are nonetheless encouraging for further studies for a better understanding of the mechanism of action by which TRPM2 inhibition induces cell death after chemotherapy treatment.

It is important to mention that the TRPM2 channel contains a NUDT9 ADP-ribose hydrolase domain which is responsible for its ADP-ribose hydrolase activity [67]. This domain allows the channel to degrade ADP-ribose, a molecule involved in cellular signaling. Since the NUDT9 domain and its enzymatic activity are important to mediate responses to oxidative stress and cellular damage, it will would be worthy to consider its potential involvement in breast cancer chemoresistance.

## 5. Conclusions

In summary, the development of TRP (transient receptor potential) channels as therapeutic targets in breast cancer represents a promising emerging revolution in treatment strategies. It is a promising approach to overcome the enormous challenge of chemotherapy resistance and improve treatment outcomes, as shown in Table 1. In this review, it was shown that inhibiting specific TRP channels, such as TRPA1, TRPC5, and TRPM2, could enhance the efficacy of conventional chemotherapeutic agents and disrupt resistance mechanisms. In fact, it was revealed that the upregulated expression of TRPA1 observed in breast cancer cells enhances resistance to ROS by triggering Ca^2+^-dependent signaling pathways that inhibit apoptosis. On the other hand, TRPA1 inhibition reduces tumor growth and improves sensitivity to chemotherapies. Moreover, TRPC5 is a potential biomarker in breast cancer chemoresistance since its overexpression in cancer cells was correlated to Adriamycin resistance. It was also shown that chemoresistant breast cancer cells overexpressing the TRPC5 transfer channel units to chemo sensitive recipient cells via extracellular vesicles leads to the development of TRPC5-mediated chemoresistance in these cells. In addition, in this review, it was proven that TRPM2 inhibition or the knock out enhances sensitivity to chemotherapy in breast cancer cells and thus increases cell death.

In another hand, studies have shown that the activation of TRPV1 and TRPV2 eventually promotes and enhances chemotherapy cytotoxicity. As indicated, TRPV1 activation was shown to enhance cisplatin oxidant effects inducing cell death via the intrinsic apoptotic signaling pathway. As mentioned in the review, the activation of the TRPV2 by its cannabidiol increases the cellular uptake of doxorubicin, which enhances the sensitivity of breast cancer cells to chemotherapy in vitro and in vivo.

A full understanding of the complex signaling cascades regulating apoptosis initiation is essential to fully comprehend the molecular complexities underlying these events. In light of current knowledge gaps, it is crucial to emphasize that, although considerable progress has been made, much of it requires rigorous validation through in vivo experiments, in line with the gold standard of research.

Finally, the permeability of TRP channels to Ca^2+^ can vary based on the specific TRP channel subtype, the cellular context, and other factors. Indeed, as well as calcium, these channels are also permeable to sodium (Na^+^) and magnesium (Mg^2+^). Thus, more deeper research on TRP channels, their ion permeability, and their roles in cellular physiology and disease are required to more deeply understand and comprehend the eventual role of each of these ions in the resistance to therapies.

## Figures and Tables

**Table 1:** Key finding on TRP Channels and breast cancer chemoresistance.

Channel	Breast Cancer Subtype	Effect	Signaling Pathway
TRPA1	PR-/ER-/HER2-	TRPA1 inhibition decreases tumor growth and increases sensitivity to carboplatin in vivo [40]	RAS-ERK/AKT/mTOR
	PR-/ER-/HER2-	The inhibition of TRPA1 increases sensitivity to doxorubicin [46]	-
TRPC5	PR+/ER+/HER2+/-	The inhibition of TRPC5 decreases doxorubicin and paclitaxel resistance in vitro and in vivo and changes drug distribution leading to the accumulation of doxorubicin in the nucleus [50]	CaMKKβ/AMPKα/mTOR
TRPV1	PR+/ER+/HER2+/-	TRPV1 activation in combination with doxorubicin increases apoptosis [56]	Intrinsic apoptotic signaling pathways
TRPV2	PR-/ER-/HER2-	TRPV2 activation via cannabidiol increases the cellular uptake of doxorubicin, which enhances apoptosis [63]	Intrinsic apoptotic signaling pathways
TRPM2	PR-/ER-/HER2-	TRPM2 inhibition increases cell death after treatment with doxorubicin and tamoxifen [66]	-
	PR+/ER+/HER2+/-	TRPM2 inhibition increases in cell death after treatment with tamoxifen and enhances the levels of DNA damage when combined with doxorubicin treatment [66]	-
